# Supplementary material for: Thermoresponsive Shape Memory Fibers for Compression Garments
Source: Polymers (Basel). 2020 Dec 15;12(12):2989. doi: 10.3390/polym12122989 (PMC7765188; doi:10.3390/polym12122989)
Supplement: Supplementary file 1 [file polymers-12-02989-s001.zip › 2020 12 14 Shape Memory Fibers - supplementary.docx]

Article

Thermoresponsive Shape Memory Blend Fiber for Compression Garments

Robert Tonndorf*, Dilbar Aibibu, Chokri Cherif

Institute of Textile Machinery and High Performance Material Technology, Technische Universität Dresden, Germany

***** Correspondence: robert.tonndorf@tu-dresden.de

Received: date; Accepted: date; Published: date

Supplementary data

| **Table S1.** Mechanical properties; mean value and standard deviation, sample size = 10. |
| --- |
| \| **Yarn Sample** \| **As-Spun** \|  \|  \|  \| **Conditioning** \|  \| **After Conditioning** \| \| \| --- \| --- \| --- \| --- \| --- \| --- \| --- \| --- \| --- \| \|  \| **Linear mass  density** \| **Tensile  strength** \| **Strain  at max. force** \|  \| **Shrinkage  at 50 °C** \|  \| **Tensile  strength** \| **Strain  at max. force** \| \|  \| **[tex]** \| **[MPa]** \| **[%]** \|  \| **[%]** \|  \| **[MPa]** \| **[%]** \| \| TPU \| 43.3 ± 0.7 \| 231.0 ± 17 \| 119.1 ± 11 \|  \| 28.4 ± 01 \|  \| 218.5 ± 11 \| 196.2 ± 13 \| \| TPU/PCL  Blend (70/30) \| 34.6 ± 0.2 \| 168.3 ± 7.8 \| 143.2 ± 8.9 \|  \| 40.8 ± 0.7 \|  \| 149.2 ± 8.9 \| 279.0 ± 19.4 \| \| TPU/PCL  Blend (90/10) \| 35.8 ± 0.9 \| 160.9 ± 11.2 \| 128.6 ± 12.2 \|  \| 27.5 ± 0.9 \|  \| 149.6 ± 9.9 \| 205.1 ± 16.3 \| \| TPU/PCL  Core-sheath \| 129.0 ± 6.2 \| 72.8 ± 3.9 \| 354.5 ± 23.2 \|  \| 8.6 ± 1.5 \|  \| 63.4 ± 5.3 \| 358.8 ± 34.0 \| \| TPU/PCL  Island-in-sea \| 103.0 ± 1.5 \| 102.6 ± 6.6 \| 150.2 ± 7.6 \|  \| 29.5 ± 0.5 \|  \| 98.9 ± 8.3 \| 250.4 ± 15.9 \| |

| **Table S2.** Thermal properties. |
| --- |
| \| **Sample** \| **Peak for Heating** \| \|  \| **Peak for Cooling** \| \| \| --- \| --- \| --- \| --- \| --- \| --- \| \|  \| **Onset** \| **Enthalpy** \|  \| **Onset** \| **Enthalpy** \| \|  \| **[°C]** \| **[J/g]** \|  \| **[°C]** \| **[J/g]** \| \| Pellets: TPU \| - \| - \|  \| - \| - \| \| Pellets: PCL \| 42 \| 64 \|  \| 32 \| 61 \| \| Yarn: TPU \| - \| - \|  \| - \| - \| \| Yarn: TPU/PCL Blend (90/10) \| 41 \| 5 \|  \| n/a \| n/a \| \| Yarn: TPU/PCL Blend (70/30) \| 43 \| 18 \|  \| 34 \| 13 \| \| Yarn: TPU/PCL Core-sheath \| 43 \| 22 \|  \| 39 \| 19 \| \| Yarn: TPU/PCL Island-in-sea \| 43 \| 14 \|  \| 41 \| 13 \| |

| **Table S3.** Shape memory properties; mean value and standard deviation, sample size = 10. |
| --- |
| \| **Yarn sample** \| **Fixity** \| **Recovery** \| \| --- \| --- \| --- \| \|  \| **[%]** \| **[%]** \| \| TPU \| 14.5 ± 1.4 \| 99.4 ± 0.8 \| \| TPU/PCL Blend (90/10) \| 35.9 ± 1.3 \| 99.6 ± 0.3 \| \| TPU/PCL Blend (70/30) \| 62.0 ± 3.3 \| 99.9 ± 0.5 \| \| TPU/PCL Core-sheath \| 37.8 ± 2.7 \| 99.9 ± 0.3 \| \| TPU/PCL Island-in-sea \| 40.2 ± 1.8 \| 99.2 ± 1.0 \| |

| **Table S4.** Force before and after stimulation at different constant strains for yarn samples with and without pre-strain fixation; mean value and standard deviation, sample size = 3. |
| --- |
| \|  \| **w/o pre-strain fixation** \| \| \|  \| **w/ pre-strain fixation** \| \| \| \| --- \| --- \| --- \| --- \| --- \| --- \| --- \| --- \| \| **Const. strain** \| **Stress pre  stimul.** \| **Stress post  stimul.** \| **ΔStress** \|  \| **Stress pre  stimul.** \| **Stress post  stimul.** \| **ΔStress** \| \| **[%]** \| **[MPa]** \| **[MPa]** \| **[MPa]** \|  \| **[MPa]** \| **[MPa]** \| **[MPa]** \| \| 10 \| 6.6 ± 0.5 \| 2.2 ± 0.1 \| -4.4 ± 0.6 \|  \| -0.1 ± 0.2 \| 1.0 ± 0.4 \| 1.1 ± 0.6 \| \| 20 \| 7.0 ± 0.2 \| 2.5 ± 0.2 \| -4.6 ± 0.4 \|  \| 0.0 ± 0.2 \| 2.4 ± 0.5 \| 2.4 ± 0.7 \| \| 30 \| 8.4 ± 0.5 \| 3.6 ± 0.4 \| -4.9 ± 0.9 \|  \| -0.1 ± 0.1 \| 2.6 ± 0.2 \| 2.7 ± 0.3 \| \| 40 \| 10.3 ± 1.0 \| 4.5 ± 0.4 \| -5.8 ± 1.5 \|  \| 0.6 ± 0.2 \| 2.9 ± 0.5 \| 2.4 ± 0.8 \| \| 50 \| 11.0 ± 0.1 \| 4.5 ± 0.1 \| -6.5 ± 0.2 \|  \| 2.0 ± 0.2 \| 3.7 ± 0.2 \| 1.7 ± 0.5 \| \| 60 \| 13.0 ± 0.9 \| 6.1 ± 0.7 \| -6.9 ± 1.6 \|  \| 3.0 ± 0.1 \| 3.5 ± 0.2 \| 0.5 ± 0.3 \| \| 70 \| 16.1 ± 2.7 \| 8.6 ± 1.4 \| -7.6 ± 4.1 \|  \| 4.9 ± 0.1 \| 4.2 ± 0.2 \| -0.8 ± 0.2 \| \| 80 \| 20.3 ± 1.0 \| 11.6 ± 0.5 \| -8.8 ± 1.5 \|  \| 8.7 ± 0.1 \| 6.0 ± 0.1 \| -2.7 ± 0.2 \| \| 90 \| 22.1 ± 1.7 \| 12.7 ± 1.0 \| -9.4 ± 2.6 \|  \| 14.5 ± 0.1 \| 9.5 ± 0.1 \| -5.0 ± 0.3 \| |

| 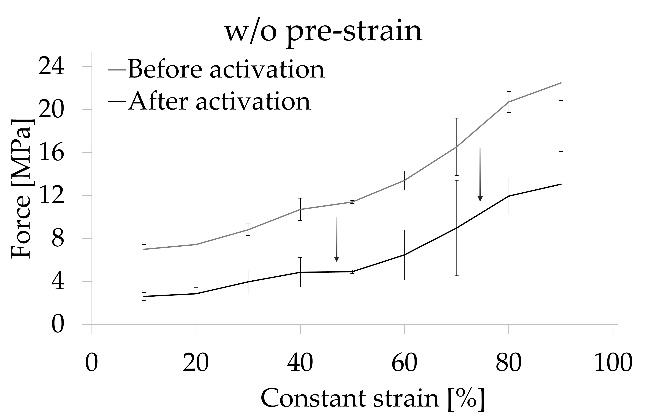  (**a**) | 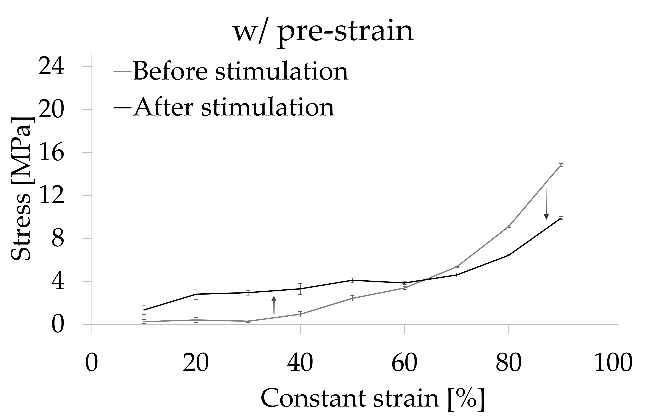  (**b**) |
| --- | --- |
| **Figure S1.** Force before and after stimulation at different constant strains for yarn samples with and without pre-strain fixation; mean value and standard deviation, sample size = 3. | |

| 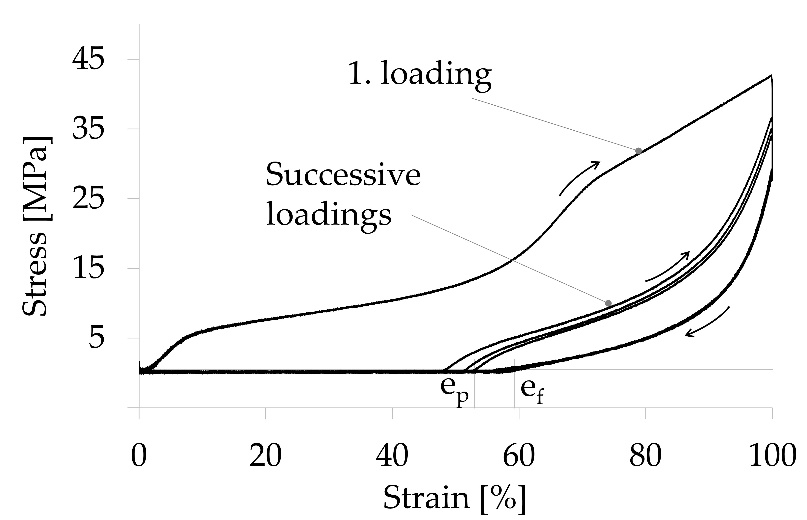 |
| --- |
| **Figure S2.** Stress-strain curve of a TPU/PCL blend 70/30 yarn sample without stimulation at 20 °C; 4 cycles (n=4), horizontal line represents force threshold of 0.6 MPa for determination of the fixed strain e_f_ and the recovered strain e_p_ |

**[Video S1]**
